# Supplementary material for: Defective Excitatory/Inhibitory Synaptic Balance and Increased Neuron Apoptosis in a Zebrafish Model of Dravet Syndrome
Source: Cells. 2019 Oct 4;8(10):1199. doi: 10.3390/cells8101199 (PMC6829503; doi:10.3390/cells8101199)
Supplement: Supplementary file 1 [file cells-08-01199-s001.pdf]

cells

MDPI

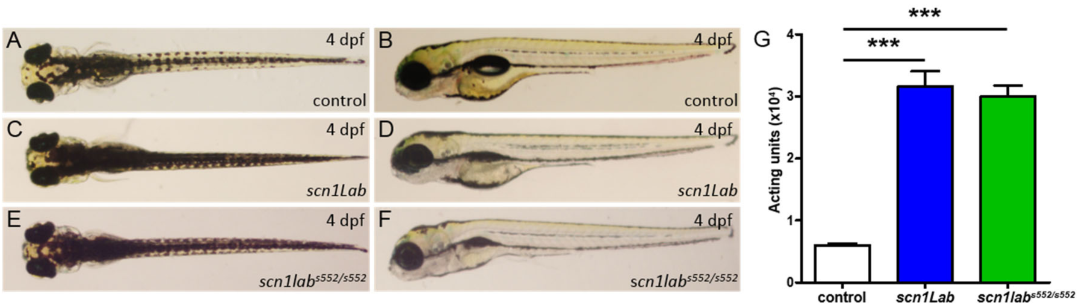

Figure S1. Comparison of *scn1Lab* morphant and *scn1Lab*$^{s552/s552}$ mutant morphology and locomotor activity. (A, C, E) Dorsal view of 4 dpf control (A), *scn1Lab* (C) and *scn1lab*$^{s552/s552}$ (E) larvae showing the hyperpigmentation of the morphant and the mutant larvae. (B, D, F) Lateral view of 4 dpf control (B), *scn1Lab* (D) and *scn1lab*$^{s552/s552}$ (F) larvae showing the impossibility for morphant and mutant larvae to inflate their swim bladder. (G) Plot of the locomotor activity of 4 dpf WT, *scn1Lab* and *scn1Lab*$^{s552/s552}$ larvae tracked during 25 min. Data were pooled from 5 independent experiments with at least 16 larvae per condition. Error bars on all graphs represent standard error mean (SEM). ***, $p <$ 0.001. p-values were determined using Kruskal-Wallis test with Dunn's multiple comparison posttest.

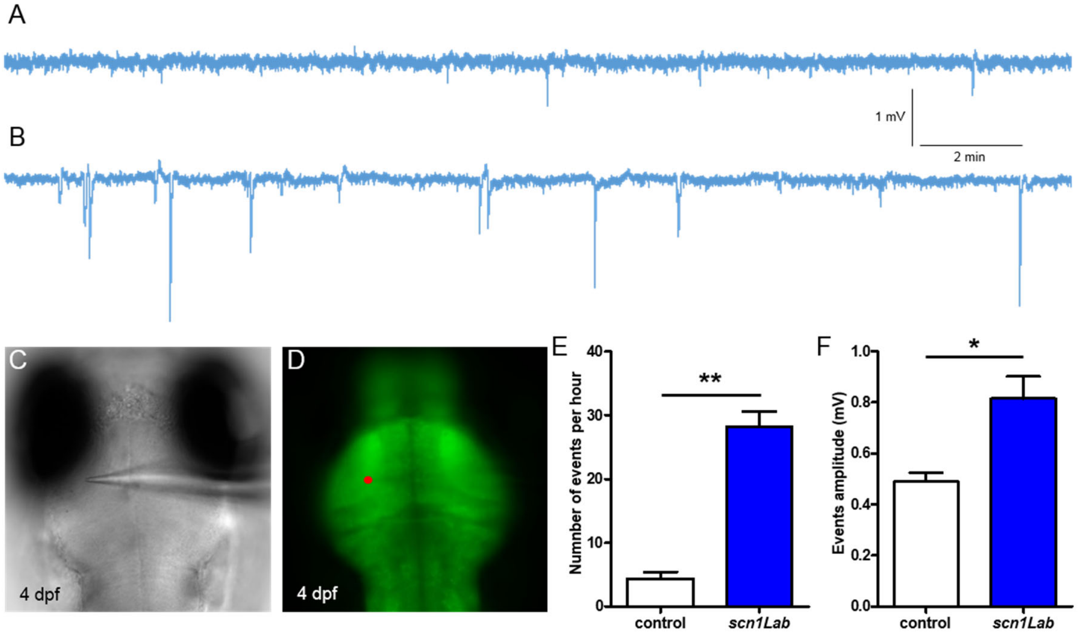

Figure S2. Neuronal activity of *scn1Lab* model. (A-B) Representative 20 min local field potential recordings in the neuropil of immobilized and paralyzed 4 dpf control (A) (N = 5) and *scn1Lab* (B) (N = 5) larvae. (C-D) Images of an immobilized and paralyzed larva during local field potential recording (C) showing the approximate localization of the electrode in the left neuropil using HuC-GCaMP5G transgenic line (D, red dot). (E) Number of events, downwards depolarization greater than 0.3 mV amplitude and lasting more than 100 s, during 1 hour recording. (F) Mean events amplitude. Error bars on all graphs represent standard error mean (SEM). *, $p < 0.05$; **, $p < 0.01$. p-values were determined using Mann Whitney test.
